# Ivory Vertebra Phenomenon as a Sign of Metastatic Dissemination in Pancreatobiliary Neoplasia: A Case Report and Literature Review

**DOI:** 10.7759/cureus.77978

**Published:** 2025-01-25

**Authors:** Madalena Santos, Mafalda Pinho, Joana Gouveia, Mariana Sousa, Lígia Peixoto

**Affiliations:** 1 Internal Medicine, Centro Hospitalar Universitário Lisboa Norte, Hospital de Santa Maria, Lisbon, PRT

**Keywords:** ampullary carcinoma, back pain, bone, ivory vertebra, pruritus

## Abstract

The bone tissue is a specialised connective tissue composed of several components that undergo constant remodelling. The balance between bone deposition and resorption is essential for maintaining a healthy bone structure. In case of a disruption in this remodelling process, which can lead to an imbalance between bone deposition and resorption, an increase in the opacity of a vertebral body may be observed in imaging studies, resulting in what is known as the “ivory vertebra sign”. This condition can be present in many diseases such as Paget's disease, lymphomas, metastatic prostate or breast tumours and osteomyelitis. We present the case of a patient with an ivory vertebra, an uncommon radiological finding, which was related to another rare disease.

## Introduction

The bone tissue consists mainly of two primary components: an organic matrix composed of collagen fibres, arranged in a highly organised structure, and an inorganic material composed of calcium phosphate that deposits within the collagen fibres and crystallises into hydroxyapatite. Overall, it's the complex interaction between the collagen framework and the hydroxyapatite crystals that is responsible for the bone’s strength and rigidity [1]. The ivory vertebra sign reflects a diffuse and homogenous increase in bone density, without changes in the adjacent intervertebral disk’s opacity or size, while preserving the size and form of the affected vertebra. Usually, the increased opacity can involve most or all of the vertebral body, giving the typical white appearance on the X-ray, hence the name “ivory” [2]. The vertebral body is composed of vascularized trabecular bone (spongy bone) [1] that can be modified due to an increase in osteoblastic activity; an imbalance between bone formation and resorption; and the replacement of the normal vascularized trabecular bone with dense, sclerotic bone. This excess in osteoblastic activity can be the result of solid metastatic tumours (more commonly breast and prostate), and also in Hodgkin’s disease, where a sclerotic response is the predominant feature [1].

In Paget's disease, the coarsening of the vertebral trabeculae and atrophy of the spongy bone can mimic an ivory vertebra. However, in Paget's disease, there's an expansion of the vertebral body contour, and it's a polyostotic disease [2]. In the case of osteomyelitis, the response to the infection can stimulate the sclerotic process and lead to a diffuse increase in bone density. The presence of erosive changes in the margins of the intervertebral disks and the involvement of several vertebrae can help in the differential diagnosis [2].

Ivory vertebra is a rare condition that can be asymptomatic or lead to nonspecific symptoms such as lower back pain, and symptoms related to the main cause, such as spinal stenosis and nerve root compression (Paget's disease), and systemic symptoms (fever, weight loss, fatigue), in case of osteomyelitis, and malignancies [1,3].

The diagnosis is made through a plain radiograph and/or computerised tomography (CT) scan that can show the diffuse sclerosis of the vertebral body. More specifically, magnetic resonance imaging (MRI) can demonstrate hypointense signals within the vertebra, which directly correlate with the degree of sclerosis of the vertebral body [4]. In cases where the diagnostic methods do not clarify its aetiology, a biopsy and culture of the vertebral body can be made in order to provide more information, either by CT-guided needle biopsy or open biopsy [5]. Nevertheless, the dense and sclerotic nature of an ivory vertebra poses a challenge to this procedure and can lead to sample inadequacy and inconclusive results [5].

## Case presentation

A 44-year-old woman presented to the Emergency Department (ED) with a three-week history of fixed lower back pain and generalized pruritus. Her past medical history was significant for obesity and essential hypertension without organ damage. The hypertension was well-controlled on a regimen of angiotensin-converting enzyme inhibitors (ACEi), calcium channel blockers (CCB), and thiazide diuretics. The patient described the lower back pain as constant, localized, worsening with movements, and relieved by non-steroidal anti-inflammatory drugs (NSAIDs). She also reported the pruritus as widespread, with no relation to xerosis, cutaneous lesions, or the use of any topical product or change in regular medication. The patient reported no significant changes in weight or appetite. On physical examination, she had icteric sclerae, scratching lesions throughout the body, and tenderness with palpation at the level of L2, with a normal spinal curvature without deformities or other significant findings. Table 1 reveals the main laboratory findings.

**Table 1:** Laboratory findings of the patient at admission The table reveals normocytic/normochromic anaemia and altered liver function tests with a slight increase in C-reactive protein. The bold values in the table indicate abnormal results.

Laboratory parameters	Value (units)	Reference value
Haemoglobin	11.9 g/dL	12.1-16.5
Haematocrit	43%	35-47
Mean corpuscular volume	88 fl	80-100
Mean corpuscular haemoglobin	28 pg	27-34
Leukocytes	10.000/mm^3^	3.500-10.000
Platelets	288.000/mm^3^	150.000-400.000
Creatinine	0.88 mg/dL	0.59-1.04
Blood urea nitrogen	34 mg/dL	17-43
Amylase	25 U/L	30-100
Aspartate aminotransferase	586 U/L	8-33
Alanine aminotransferase	942 U/L	19-25
Gamma-glutamyl transferase	657 U/L	5-40
Total bilirubin	3 mg/dL	0.1-1.2
Direct bilirubin	2.12 mg/dL	<0.3
Lactate dehydrogenase	440 U/L	125-220
C-reactive protein	1.58 mg/dL	0.8-1

**Figure 1:**
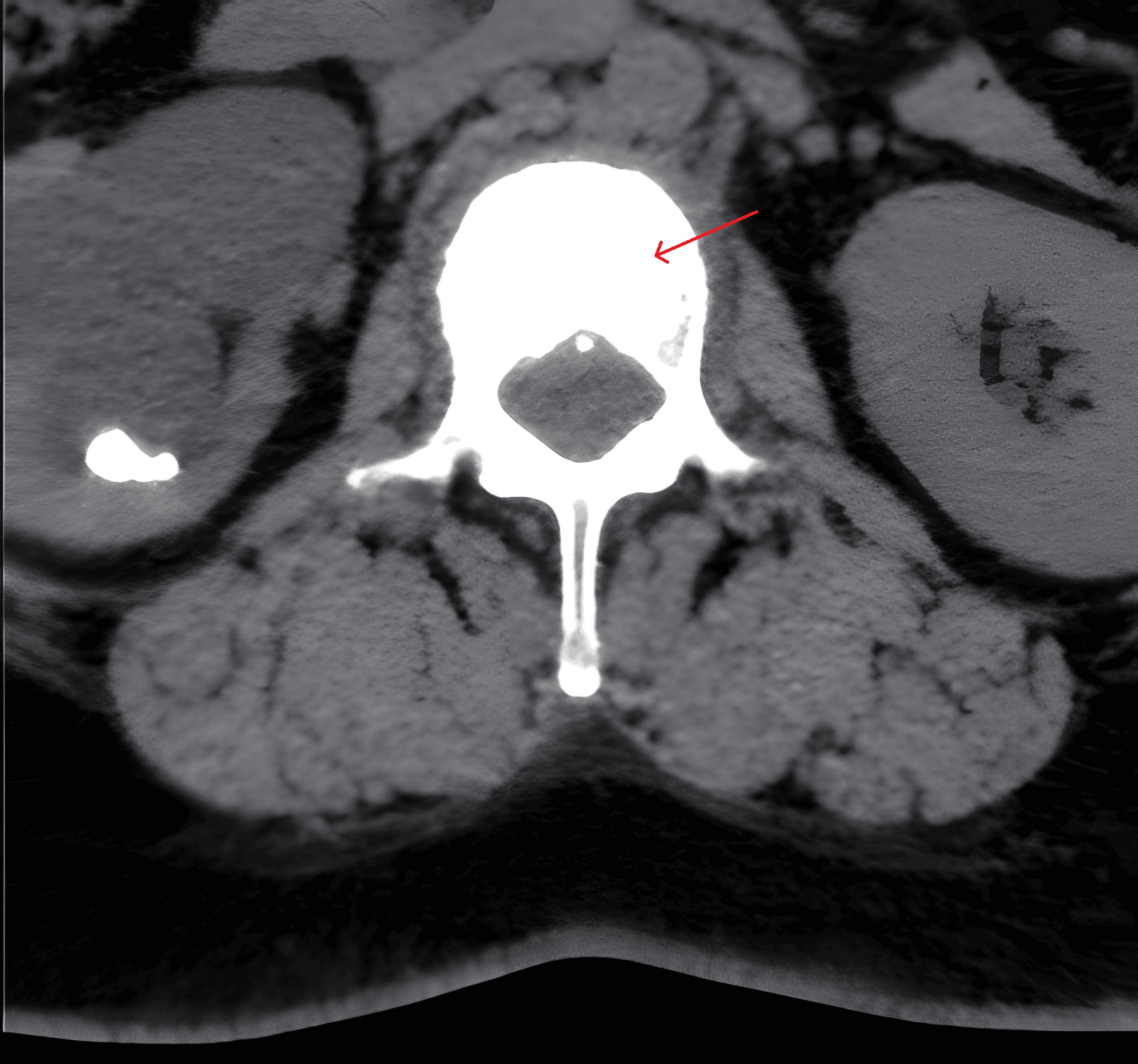
Axial image demonstrating an ivory vertebra at L2 (arrow), with increased opacity of the vertebral body.

A non-contrast abdominopelvic CT scan was performed, which described bile duct dilatation with two stones located in the papilla, pancreaticoduodenal lymphadenopathy, and an ivory vertebra sign in L2 (Figure 1).

**Figure 2:**
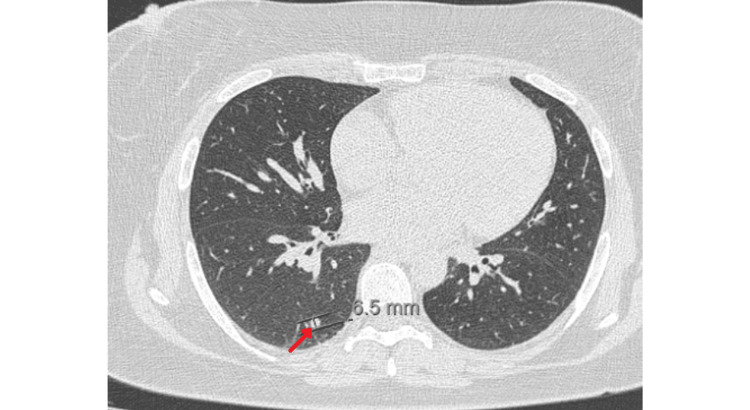
Axial image showing pulmonary nodules in the left lower lobe, with the largest measuring 6.5 mm in size (arrow).

The patient was then admitted to a medical ward for an etiological investigation. During hospitalization, a chest CT scan revealed peripheral nonspecific infracentimetric pulmonary micronodules without associated adenopathy (Figure 2). Bronchoscopy showed no abnormalities. The biopsies and bronchoalveolar lavage were negative for neoplasm.

**Figure 3:**
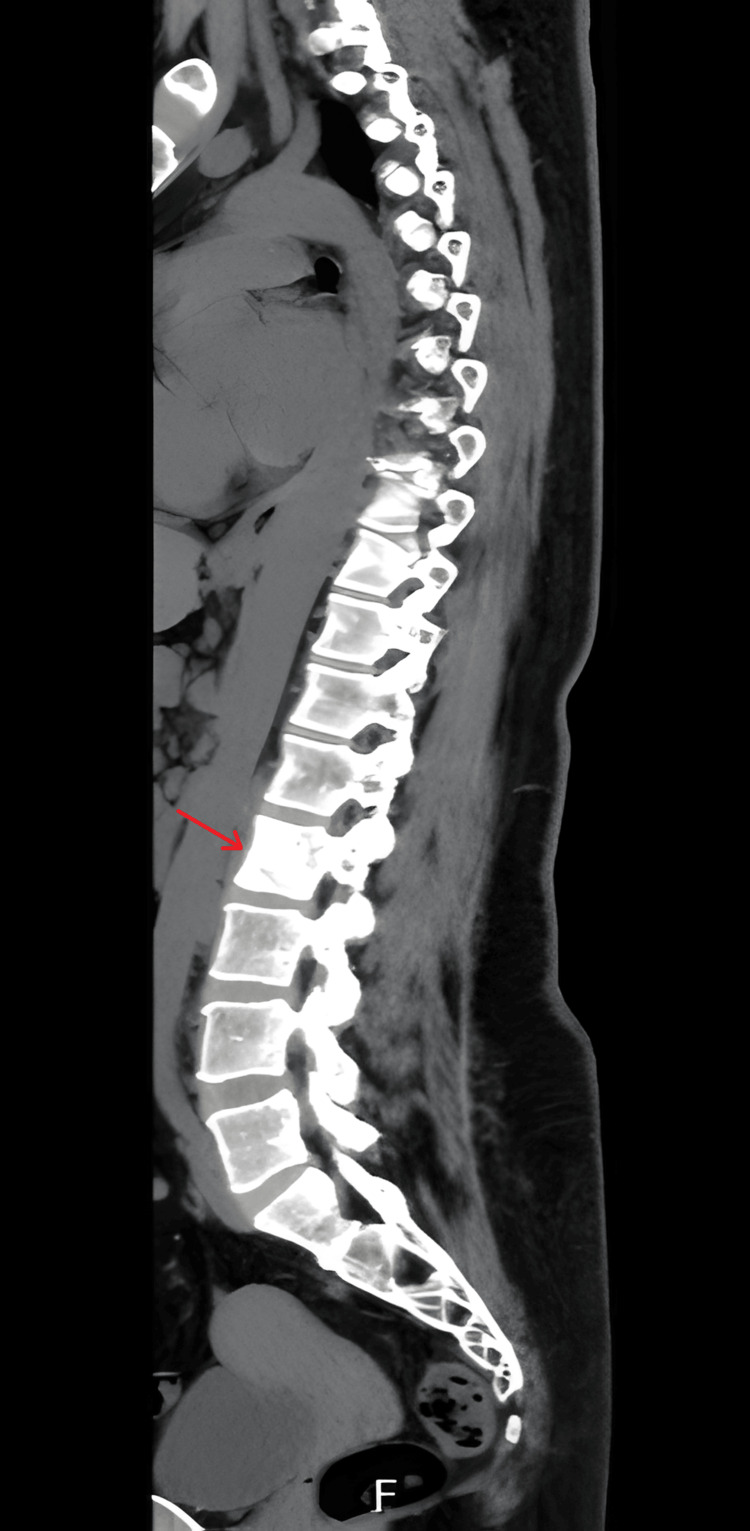
Sagittal image of the vertebral column, with the arrow highlighting the ivory vertebra sign at L2.

A CT scan of the vertebral column revealed a sclerotic lesion in L2, without spinal stenosis or nerve root compression, and without further involvement of other vertebrae (Figure 3).

**Figure 4:**
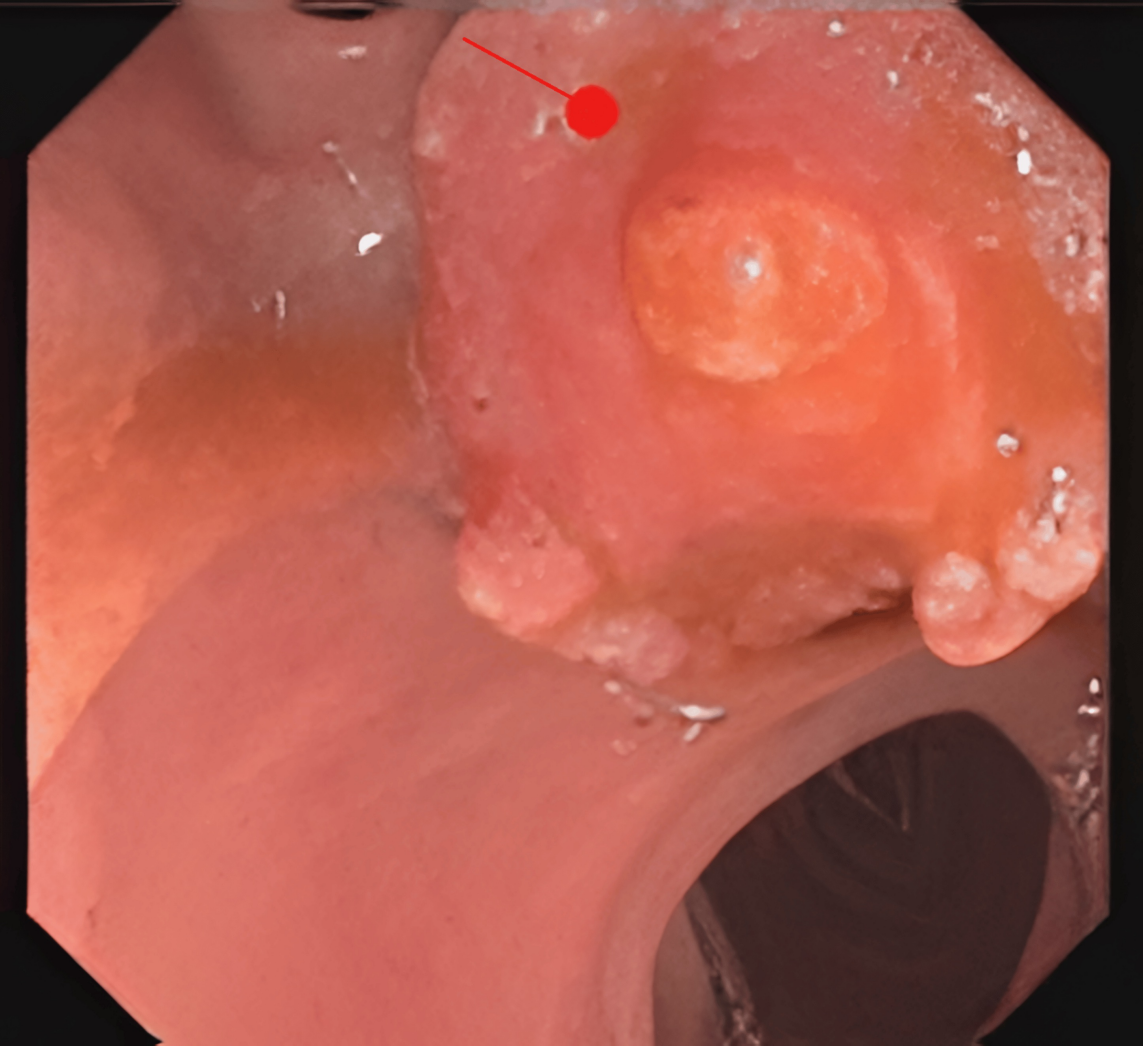
Image of the ulcerated tumor in the papilla of Vater (arrow) obtained through endoscopic retrograde cholangiopancreatography (ERCP). Image obtained through ERCP showing an ulcerated, exophytic, ulcerofungating tumour in the duodenal lumen. The tumour eccentrically engulfs the ampulla of Vater orifice, with minimal involvement of the intra-ampullary region (arrow).

The patient underwent an endoscopic retrograde cholangiopancreatography (ERCP) that revealed dilation of the common bile duct and the pancreatic duct (double duct sign) with papillary stenosis caused by an ulcerated and exophytic tumour growing in the ampulla of Vater (Figure 4). No obstructive calculi were found. During the procedure, two stents were placed (one in the stenotic area and the other in the pancreatic duct), and biopsies were performed without related complications.

**Figure 5:**
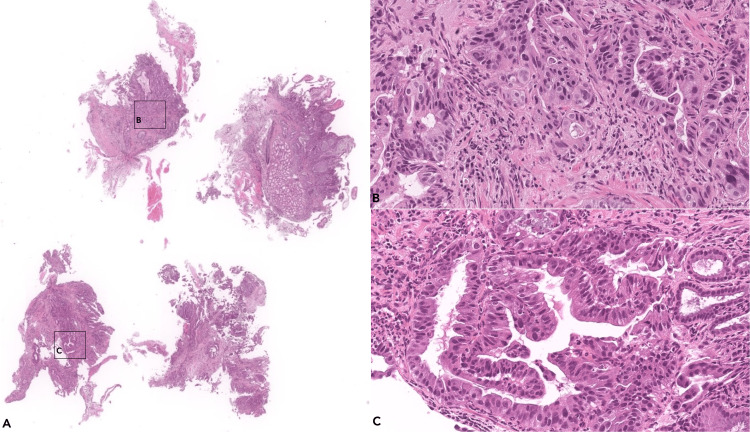
Histology of the ulcerated tumor in the papilla of Vater Fragments of mucosa from the anatomical region showing infiltration by a gland-forming (tubular) adenocarcinoma consisting of relatively small glands separated by a desmoplastic stroma (A). The glands are predominantly small and lined by a single layer of pleomorphic cuboidal to low columnar cells, exhibiting a pancreatobiliary pattern (inset B); in some other areas, the glands are lined by tall columnar cells with elongated, pseudostratified nuclei, reflecting an intestinal pattern (inset C). These findings are consistent with a tubular adenocarcinoma with mixed features, with a predominant pancreatobiliary pattern.

The biopsy showed infiltration by a moderately differentiated tubular adenocarcinoma with mixed features, with a predominant pancreatobiliary pattern (Figure 5). The biomarker analysis by next-generation sequencing (NGS) detected clinically significant alterations in the KRAS gene: c.35G>A p. (Gly12Asp) in exon 2 (with an allelic frequency of 11.67%). No other variants, rearrangements, or copy number alterations were detected. The tumour was microsatellite stable (MSS).

During the patient's stay in the medical ward, all the main conditions related to the ivory vertebra sign were excluded, including infectious causes, Paget's disease, and haematological malignancies. A complete oncology screening with a body CT scan and mammography was performed to reach a definitive diagnosis. To complement the findings described above and confirm if the pulmonary nodules and ivory vertebra were related to the ampullary tumour, a fluorodeoxyglucose-positron emission tomography (FDG-PET) was performed. It revealed metabolically active disease in the image corresponding to the ampullary neoplasm, with associated high expression in L2, multifocal abdominal lymph nodes, and the pulmonary nodules, all suspicious of a metastatic nature. Ampullary adenocarcinoma with a predominant pancreatobiliary pattern was further staged as IV, with pulmonary, lymph node and bone metastasis. The patient underwent palliative chemotherapy with cisplatin and gemcitabine, but unfortunately, her condition continued to deteriorate, and the disease progressed, culminating in her demise approximately 18 months after the initial diagnosis.

## Discussion

Tumours of the ampulla of Vater are significantly rare, representing about 0.2% of gastrointestinal tumours [6]. Peak incidence occurs between 60 and 80 years of age, with a mild male predominance [7]. They usually present with jaundice, diarrhoea, and melena, and progress with metastasis to lymph nodes and adjacent organs such as the liver and, in more advanced cases, to the lungs [8]. The diagnosis can be made by endoscopic ultrasound (EUS), ERCP, and fine-needle aspiration cytology (FNAC), while the staging of the tumour can be done with the use of a full-body CT scan [8].

This case report describes a young female patient who presented to the ED with a history of lower back pain, which was the result of the secondary lesion in L2, and with generalized pruritus, which can be related to the cholestatic abnormalities observed in the laboratory tests. The absence of the typical symptoms described above most likely delayed the definitive diagnosis, leading to the identification of the disease when the tumour had already involved other organs. Despite the diagnosis of ampullary adenocarcinoma and the exclusion of other conditions related to the ivory vertebra, it was extremely important to investigate if the multiple pulmonary nodules and the ivory vertebra itself were the result of a metastatic process.

Given the high risk of procedure-related complications and the high likelihood of inconclusive and/or inadequate results from biopsy of both sites, it was decided to first perform a PET scan that could confirm the presence of the ampullary adenocarcinoma and its metastatic spread to other organs [9]. Therefore, the PET scan results were extremely important in the staging of the ampullary adenocarcinoma and allowed for prompt treatment of the tumour with chemotherapy [10].

The treatment for tumours of the ampulla of Vater consists of a radical surgical resection of several structures with lymphadenectomy, more specifically a pancreaticoduodenectomy, in case there's no other organ involvement [11]. Complete surgical resection represents the most effective treatment modality currently available [12]. In this clinical scenario, the extensive metastatic involvement was the primary rationale for initiating systemic chemotherapy.

Recent literature has documented cases of ampullary carcinomas with bone involvement [7]. Additionally, pancreatic tumours with metastasis to the bone, presenting with the ivory vertebra sign, have been reported [13]. However, to date, there have been no documented cases of ampullary carcinoma with metastatic involvement of the vertebral body manifesting as an ivory vertebra sign. This underscores the critical importance of conducting a comprehensive investigation of a patient's medical history and maintaining a high index of suspicion for atypical metastatic patterns.

Ampullary adenocarcinoma, despite its rarity, carries a poor prognosis. The five-year survival rate ranges from 41% to 45% for locally confined disease, dropping precipitously to 4% to 7% for metastatic cases [11]. Notably, tumours exhibiting pancreaticobiliary immunohistochemical subtypes demonstrate an even more unfavourable prognosis [11].

## Conclusions

This case highlights the rarity and diagnostic challenges associated with both the ivory vertebra sign and metastatic ampullary adenocarcinoma. The ivory vertebra sign, characterized by a uniformly increased opacity of a vertebral body on radiographs with the normal appearance of adjacent vertebral bodies, is an uncommon finding that can be easily overlooked. Similarly, ampullary adenocarcinoma with metastatic involvement is infrequently reported in medical literature.

This case illustrates the importance of a thorough diagnostic workup when encountering such rare presentations, and it emphasizes the crucial role of advanced imaging techniques, particularly PET scans, in confirming the diagnosis and extent of metastatic spread. By showcasing the utility of PET in detecting and characterizing both the primary tumour and its metastases, this case provides valuable insights for clinicians facing similar diagnostic challenges, potentially improving patient management and outcomes in these uncommon scenarios.
